# Osteopontin—A Master Regulator of Epithelial-Mesenchymal Transition

**DOI:** 10.3390/jcm5040039

**Published:** 2016-03-23

**Authors:** Anai N. Kothari, Matthew L. Arffa, Victor Chang, Robert H. Blackwell, Wing-Kin Syn, Jiwang Zhang, Zhiyong Mi, Paul C. Kuo

**Affiliations:** 1 Loyola University Medical Center, Department of Surgery, 2160 S First Ave, Maywood, IL 60153, USA; ankothari@lumc.edu (A.N.K.); marffa@luc.edu (M.A.); vchang@luc.edu (V.C.); rblackwell@lumc.edu (R.H.B.); zhmi@luc.edu (Z.M.); 2 Burn and Shock Trauma Institute, Loyola University Chicago, 2160 S First Ave, Maywood, IL 60153, USA; 3 Division of Gastroenterology and Hepatology, Medical University of South Carolina, Charleston, South Carolina, USA; synw@musc.edu; 4 The Oncology Institute, Cardinal Bernardin Cancer Center, Loyola University Medical Center, Maywood, IL 60153, USA; jzhang@luc.edu

**Keywords:** osteopontin, epithelial-mesenchymal transition, cancer-associated fibroblasts, tumor microenvironment, tumor metastasis, fibrosis

## Abstract

Osteopontin (OPN) plays an important functional role in both physiologic and pathologic states. OPN is implicated in the progression of fibrosis, cancer, and metastatic disease in several organ systems. The epithelial-mesenchymal transition (EMT), first described in embryology, is increasingly being recognized as a significant contributor to fibrotic phenotypes and tumor progression. Several well-established transcription factors regulate EMT and are conserved across tissue types and organ systems, including TWIST, zinc finger E-box-binding homeobox (ZEB), and SNAIL-family members. Recent literature points to an important relationship between OPN and EMT, implicating OPN as a key regulatory component of EMT programs. In this review, OPN’s interplay with traditional EMT activators, both directly and indirectly, will be discussed. Also, OPN’s ability to restructure the tissue and tumor microenvironment to indirectly modify EMT will be reviewed. Together, these diverse pathways demonstrate that OPN is able to modulate EMT and provide new targets for directing therapeutics.

## 1. Introduction

Osteopontin (OPN) was first described by Senger *et al.* in 1979 as a phosphoprotein secreted into growth medium by transformed epithelial cells [1]. OPN plays an important functional role in many biologic processes and systems [2]. Although named for its role in non-collagenous bone matrix formation, the glyco-phosphoprotein is expressed by numerous cell types including osteoclasts, osteoblasts, epithelial cells, lens cells, pericytes, fibroblasts, hepatocytes, tubular cells, and vascular smooth muscle cells [3].

A wide variety of cell types express OPN and, as a result, it plays several physiologic roles including the regulation of inflammation and immunity. OPN plays a role in both acute and chronic inflammation by modulating effector cell types including macrophages and T-cells. For example, macrophages act as both a target and source for OPN: several cytokines are capable of inducing OPN expression in macrophages and OPN is an effective chemoattractant [4]. OPN is also highly expressed in active T cells and is capable of promoting cell mediated immune responses [5]. Non-specifically, OPN can regulate immune suppression, angiogenesis in the setting of stress response, cell adhesion and chemotaxis, and cellular motility [6]. These latter functions are important for the pathologic influences of OPN.

OPN is highly implicated in fibrosis, tumorigenesis, and cancer metastasis. This is largely a function of the diverse cellular repositories for OPN across tissue types. OPN is mostly highly expressed in bone (osteoclasts, osteoblasts), breast (epithelial cells), kidney (epithelial cells, tubular cells), skin, nerves, and the liver. It is also present in plasma, urine, milk, and bile [6]. Studies show that OPN is critical for the progression of liver, renal, pulmonary, and cardiac fibrosis [7,8,9]. Further, OPN is also important for tumor initiation and invasion in liver, gastric, colorectal, and lung cancer [10,11]. In both fibrosis and cancer, the mechanisms through which OPN exerts its effect are incompletely understood. The link between OPN and the epithelial-mesenchymal transition (EMT), however, appears to be a significant molecular explanation for how OPN influences both of these processes.

EMT was first noted by work from Elizabeth Hay in 1980s in the setting of embryogenesis [12]. EMT is defined by epithelial cells taking on a mesenchymal phenotype characterized by loss of apical-basal polarity, increased cellular motility, and reorganization of their cytoskeleton [13]. Broadly, there are 3 major types of EMT: type 1 referring to embryogenesis; type 2 referring to wound healing; type 3 referring to cancer and metastases [14].

In each type of EMT, several well-established transcriptional regulators, including ZEB, SNAIL, and TWIST, can repress E-cadherin and activate key mesenchymal genes including N-cadherin (cell-cell adhesion), vimentin (an intermediate filament protein that regulates cell motility), and fibronectin 1 (cell growth and migration) [15]. Beyond these, additional regulatory factors including transcription factors, miRNAs, and the microenvironment all play integral roles in transitioning cells from an epithelial to mesenchymal phenotype [16].

**Figure 1:**
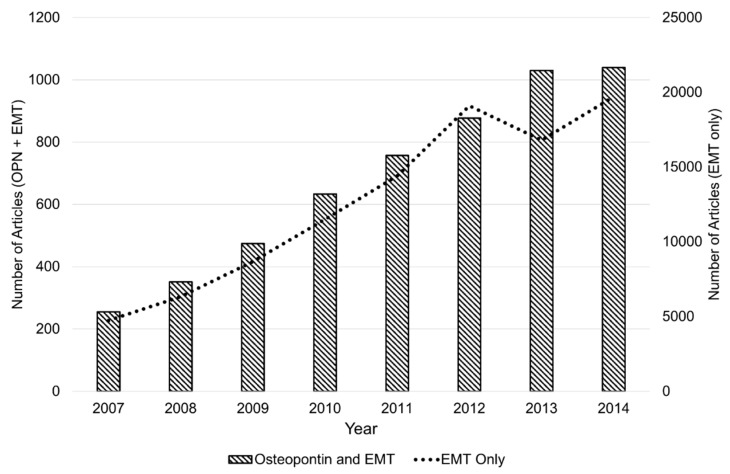
The appearance of “osteopontin” and epithelial-mesenchymal transition in the same peer-reviewed scientific article over time (per year), beginning in 2007. Data on number of articles obtained using Google Scholar search with terms “osteopontin (OPN) + epithelial-mesenchymal transition (EMT)” *vs.* “EMT” only, excluding patents and citations.

Increasingly, OPN is being recognized as a key regulator of EMT through each of these diverse molecular pathways—particularly in types 2 and 3 EMT (Figure 1). This review will focus on the current progress of understanding the emerging role of OPN as an integral component of initiating EMT in multiple pathologic settings. In particular, the clinical implications, where applicable, of OPN’s regulation of EMT will be highlighted.

## 2. OPN Signaling and Transcriptional Regulation of EMT

OPN plays an important regulatory role in the expression of many well-known activators of the epithelial-mesenchymal transition. It is important to note that activation of EMT does not always translate to cancer progression and cells within a tumor can be at different stages of EMT [13,17]. Nevertheless, the interplay between OPN and several common EMT pathways (TWIST, ZEB, SNAIL) is critical to activation and progression in several settings. These relationships will be reviewed in this section and are presented in Table 1.

### 2.1. Overview of Twist and EMT

Twist-related protein 1 (Twist1) and Twist-related protein 2 (Twist2) are helix-loop-helix transcription factors encoded by the TWIST1 and TWIST2 genes, respectively. Twist1 was discovered in studies examining *Drosophila melanogaster* embryology and participates in type 1 EMT in several species [18]. Overexpression of Twist1 can lead to solid tumor formation and metastatic disease in cancer. Twist2 is also important in embryologic development with some overlap in function with Twist; however, does have a distinct expression profile. In cancer, this manifests as differences in role depending on tumor type [19].

Both Twist1 and Twist2 can promote tumor progression through strong induction of EMT programs. Twist1 was first implicated in cancer as an important factor in the initiation of tumors by suppressing apoptosis through the ARF/MDM2/p53 pathway [20]. More recent work, using conditional knockdown models at various phases of tumor growth, demonstrates that Twist1 is critical for tumor initiation in skin tumorigenesis. At tumor initiation, Twist1 appears to exert its regulatory function either directly or indirectly through p53, not by activating EMT [21].

In tumor growth and metastasis, however, the interaction between Twist1 and EMT is paramount. Twist1 overexpression is associated with increased invasiveness and metastatic potential in several cancers including human glioma, neuroepithelial malignancies, hepatocellcular carcinoma, breast, esophageal, and others [22,23,24,25,26]. Mechanistically, Twist1 appears to confer these properties on tumor cell populations through EMT. In human breast cancer cell lines, the absence of Twist causes previously metastatic breast cell lines to lose the ability to form lung metastases. Twist appears to regulate the metastatic phenotype by inducing EMT, evidenced by loss of cell-cell contact and acquired fibroblastic morphology with ectopic Twist expression. At the molecular level, Twist expression leads to repression of E-cadherin transcription and the consequent initiation of EMT in these cells [25]. Similarly, in hepatocellular carcinoma (HCC), overexpression of Twist is correlated with decreased E-cadherin expression based on data from patient-derived tissue arrays. Also, highly metastatic HCC cell lines have higher Twist expression profiles than low metastatic cell lines. Ectopic Twist expression in low metastatic cell lines increases their invasion and metastatic potential through decreased E-cadherin expression and subsequent EMT [24]. In glioblastoma, Twist modulates the E- to N-cadherin switch using CXCL12-induced signaling. Treatment with CXCL12 results in significant upregulation of Twist protein levels and promotion of EMT. In mice models, the activation of EMT creates an aggressive and invasive tumor phenotype [27].

Another mechanism for Twist to impact tumor behavior is by activating EMT to enrich cancer stem cell populations. Both Twist1 and Twist2 are overexpressed in breast cancer cells exhibiting a stem-like phenotype (CD44high/CD24low). More directly, ectopic expression of Twist1 leads to dedifferentiation into cancer stem cells through downregulation of CD24 following EMT. Twist2 also appears to have a functional role in activating EMT to regulate stemness in ovarian cancer cells [28,29]. As a result, Twist1 and Twist2 can generate a population of self-renewing cancer stem cells through EMT.

### 2.2. OPN, Twist, and EMT

It is clear that Twist represses E-cadherin transcription to activate EMT. However, understanding the upstream and downstream factors that impact the Twist/E-cadherin system may provide important therapeutic insight. OPN and Twist upregulation were first linked in the setting of osteogenesis. Using an osteoblast-like cell line, differential expression of Twist was shown to modify osteoblastic differentiation and, at increasing levels of Twist, OPN mRNA levels also increased [30]. This physiologic interplay between OPN and Twist extends to pathologic conditions, including cancer and fibrosis.

In breast cancer cell lines, OPN leads to an increase in expression of several EMT-related transcription factors including Twist, Snail, and Slug. OPN overexpression results in serine phosphorylation of Twist and binding to the B lymphoma Mo-MLV insertion region 1 homolog (Bmi-1) promoter The OPN/Twist/Bmi-1 pathway ultimately activates EMT programs in these cell lines [31]. The phosphorylation of Twist by OPN appears to occur through a MAPK pathway [32]. Another connection between OPN and Twist in breast cancer is through BMP-2. Compared to infiltrating breast carcinomas without microcalcifications, infiltrating breast carcinoma with microcalcifications upregulate BMP-2 and OPN, allowing them to acquire a mesenchymal characteristics and osteoblast-like phenotype [33]. This may occur through Twist modification by BMP-2 leading to activation of EMT [34]. Similarly, OPN is able to mediate EMT in HCC models through its regulation of Twist. OPN overexpression activates a PI3K-AKT-Twist pathway leading to EMT and HCC metastases [35].

OPN also functions through hypoxia-inducible factor-1α (HIF-1α) upregulation to induce EMT through Twist activation. HIF-1α is stabilized by intratumoral hypoxia and can cause tumor progression. HIF-1α can regulate the expression of Twist by binding directly to a segment in Twist’s proximal promoter. Binding to the hypoxia-response element by HIF-1α results in upregulation of Twist activity and promotes EMT [36]. In ovarian cancer, OPN expression increases the intratumoral concentration of HIF-1α through PI3K-AKT mediated signaling. A similar upregulation of HIF-1α is also seen in gastric cancer. In both ovarian and gastric cancer, overexpression of OPN leads to increased metastasis and invasiveness of tumors in *in vivo* models [37,38].

OPN also plays a role in maintaining the stemness of HCC through increasing the expression of HIF-1α. Unlike in ovarian and gastric cancer, induction of HIF1-α occurs through integrin α_v_β_3_ binding and activation of NF-κB [39]. To date, no studies have shown OPN-mediated increases in HIF-1α result in Twist-dependent EMT and formation of cancer stem cells. However, based on existing literature, it appears this is a candidate signaling cascade linking OPN, EMT, and cancer cell stemness.

Recently, OPN has been demonstrated to be both a biomarker and key mechanistic contributor to metastatic disease in colorectal cancer. In postoperative patient plasma, increased OPN levels are associated with the development of future metastases. The role of OPN in this process appears to be mediated by its interaction with Twist leading to enhanced cell migration, increased invasion, and decreased cell-cell adhesion in ectopically high OPN secreting cell lines [40].

**Figure 2:**
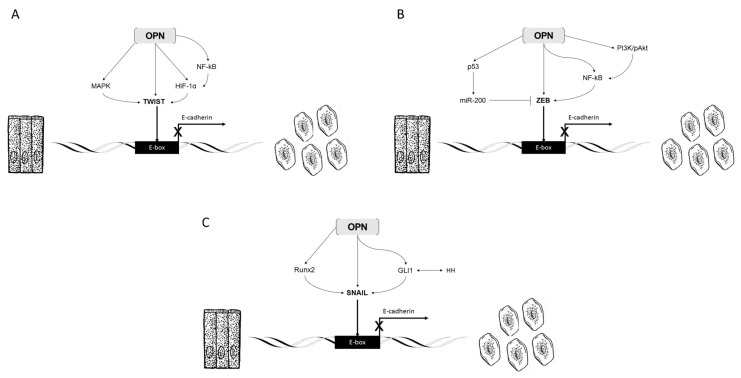
Overview of key EMT pathways regulated by osteopontin directly and indirectly. (**A**) Regulation of TWIST and EMT by OPN; (**B**) Regulation of zinc finger E-box-binding homeobox (ZEB) and EMT by OPN; (**C**) Regulation of SNAIL and EMT by OPN.

These studies together show that OPN plays an important role in the activation of Twist-dependent gene expression. OPN is positioned as an upstream and critical regulator of EMT (Figure 2A).

### 2.3. Overview of ZEB and EMT

Zinc finger E-box-binding homeobox (ZEB) is a family of zinc finger transcription factors that plays a major role in both normal development and disease. There have been two homologous ZEB proteins found in vertebrates, ZEB1 and ZEB2. All ZEB proteins have two zinc finger clusters that bind to ZEB boxes of the regulatory regions of target genes [41]. Mutations in ZEB genes have been shown to cause severe syndromic malformations, highlighting their importance in early embryonic developmental processes including those involving EMT. As a result, recent studies have found that ZEB1 and ZEB2 play critical roles in repressing E-cadherin leading to the activation of EMT programs [42].

ZEB1 and ZEB2 are known to be mediated by signaling cascades triggered by TGFβ, NF-κB, MAPK/ERK, and HIF-1α, amongst others, in activated tumors resulting in EMT [43]. In well-differentiated areas of carcinomas, ZEB1 is expressed at low levels, but high ZEB1 expression has been found to be inversely correlated to the expression of E-cadherin in dedifferentiated, fibroblastic-like cells at the periphery of invading tumors [44]. Specifically, high ZEB1 expressing cells have been found in invading endometrial, colorectal, lung, breast, prostate, gallbladder, hepatocellular, and pancreatic carcinomas [45].

In addition, stromal cells in colorectal, breast, lung, and bladder carcinomas have been found to be ZEB1-positive, suggesting that paracrine ZEB1 signaling could be responsible for E-cadherin down-regulation in parts of the tumor. Interestingly, ZEB2 has been shown to be highly expressed in E-cadherin-positive epithelial cells in the esophagus, stomach, colon, rectum, hepatocytes, and renal tubule, and subsequently down-regulated during transition to carcinomas [46,47,48]. This provides further evidence that both ZEB1 and ZEB2 can exert transcriptional regulation of EMT.

### 2.4. OPN, ZEB, and EMT

OPN-related signaling cascades interact with ZEB family members leading to modulation of EMT, both directly and indirectly. OPN is a potent activator of NF-κB and therefore can regulate NF-κB/ZEB-dependent EMT. In liver fibrosis, OPN is a key regulator of the deposition of type 1 collagen. This occurs through OPN binding to a cell surface integrin (α_v_β_3_) and activating the PI3K/pAkt/NF-κB-signaling pathway. Activation of NF-κB, in this setting, is a direct downstream effect of OPN [49]. In the breast cancer cell line MCF10A, activation of NF-κB leads to the adoption of an EMT-like phenotype. This is related to an association between activation of NF-κB and increased expression of both ZEB1 and ZEB2 [50]. The connection between NF-κB activation and ZEB1 extends beyond mammary tumors [51]. In *Helicobacter pylori* infection, gastric epithelial cells undergo EMT in response to chronic exposure. Infection with H. pylori results in recruitment and activation of NF-kB leading to transactivation of ZEB1, explaining the mechanism through which EMT is induced by the bacteria [52].

OPN can also regulate ZEB-related EMT through non-NF-κB pathways. One of the most commonly cited regulatory elements of ZEB1 and ZEB2 are microRNAs (miRNA) from the miR-200 family. Members of this family suppress tumor invasiveness and metastasis by inhibiting ZEB1 and ZEB2 initiated EMT. Interestingly, p53 appears to exert anti-tumor and anti-invasive properties in part through upregulation of miR-200 family members [53]. OPN and p53 interact in many settings, suggesting a role for OPN in this miR-200/ZEB feedback loop. A summary of these interactions is shown in Figure 2B.

### 2.5. Overview of Snail and EMT

Snail was first discovered in 1984 and found to be essential for the development of the mesoderm and neural crest. Absence of Snail during embryonic development is lethal. The Snail family members are characterized as zinc-finger transcription factors that bind to a CAGGTG motif, known as the E-box. Much like TWIST and ZEB, Snail has also been shown to be a strong repressor of E-cadherin and a key transcription factor promoting EMT [54]. In particular, Snail is present in invasive human carcinoma cell lines and tumors where E-cadherin expression has been lost, suggesting that the same molecules are used to trigger EMT during embryonic development and tumor progression.

In addition to down-regulating E-cadherin, Snail has been associated with the down-regulation of other epithelial factors including Claudins, Occludins, and Muc1 [55,56]. Snail is also responsible for up-regulating genes associated with mesenchymal and invasive traits, such as fibronectin and MMP-9. The Snail family is the most widely studied of the major EMT program activators. As a result, numerous signaling pathways are implicated in the induction of Snail1 and Snail2 expression, including TGF-β, integrin-link kinase (ILK), phosphatidylinositol 3-kinase (PI3-K), MAPK/ERK, glycogen synthase kinase 3-β (GSK-3β), Raf kinase inhibitor protein (RKIP), NF-κβ, and Tat-interacting protein (TIP30) [57,58]. Environmental signals, such as hypoxia and stress, are also known inducers of Snail1 and Snail2. The various molecular pathways that converge on Snail1 and Snail2 are well-described in several other reviews [41,58,59].

### 2.6. OPN, Snail, and EMT

OPN can modify Snail1 and Snail2 signaling to modulate EMT in several settings. Blockade of OPN using an OPN-specific aptamer results in decreased expression of Snail in both a breast and hepatocellular carcinoma model [31,60]. Ectopic OPN expression can induce cell migration through overexpression of Snail and subsequent E-cadherin repression [40].

Several specific signal transduction pathways appear to regulate the interplay between OPN and Snail. Sonic hedgehog or hedgehog (HH) is critical in development and is mediated by the GLI family of transcription factors, including GLI1. HH-GLI1 signaling is implicated in progression and metastases of several malignancies [61,62,63]. One mechanism is Snail1 induction by GLI1, with resultant promotion of EMT as demonstrated in skin cancer and brain tumor models [64,65]. The link between OPN and HH pathways is through GLI1. OPN and GLI1 are co-regulated, with evidence that each have promoter binding sites for each other [66]. Clinically important relationships between OPN and GLI1 exist in nonalcoholic steatohepatitis (NASH) and melanoma, both of which demonstrate mechanisms OPN is able to modulate the EMT in the setting of fibrosis and cancer [67,68].

Another pathway regulated by OPN that converges on Snail is through Runx2. In patient-derived breast cancer clinical specimens, patients with combined Runx2/Snail positivity have shortened disease-free survival when compared to those with either Runx2-only or Snail-only positivity. This is potentially attributable to Runx2/Snail positive tumors having increased metastatic potential mediated by EMT [69]. OPN interacts directly with Runx2 signaling in breast cell lines, linking OPN to Snail-dependent EMT [70,71]. Further investigation of shared OPN and Snail pathways can be a suitable strategy for understanding and combating cancer metastasis.

Recent studies have elucidated another mechanism by which OPN induces EMT in hepatitis C virus (HCV)-induced liver disease. Elevated serum and liver OPN levels in patients with chronic hepatitis C have been correlated with advanced liver fibrosis and a reduced response to anti-viral therapy [72]. HCV induces OPN expression and, in turn, upregulates AP-1 and Sp1 transcription factors [73]. AP-1 is a downstream target of MAPK and can induce Snail-dependent EMT [74].

The pathways leveraged by OPN to control EMT through Snail transcriptional regulation are shown in Figure 2C.

## 3. Section 2: OPN Modifies the Microenvironment to Regulate EMT

A major regulatory component of EMT comes from the complex interaction between the cell and its surrounding environment comprised of other cells, stromal elements, infiltrating immune mediators, cytokines, microRNA, exosomes, and other factors. In a healing wound, this is broadly termed the tissue microenvironment whereas in malignancy, it is the tumor microenvironment. In each setting, the microenvironment can influence the progression of physiologic and pathologic processes.

OPN is a known mediator of the composition of the microenvironment, including playing a functional role in cell-microenvironment interplay. There is mounting evidence that OPN is able to modify the tissue and tumor microenvironment to support EMT. In the following section, the role OPN plays in influencing the structure of the microenvironment will be discussed; more specifically, how OPN regulates EMT using the microenvironment.

### 3.1. Myofibroblast Activation and Fibrosis

Fibrosis is the abnormal wound healing response characterized by the excessive deposition of extracellular matrix (ECM) proteins such as collagen and fibronectin [75]. It is seen in organs such as the lung, liver, heart, and kidney, and as the fibrosis progresses, it can cause organ failure [8,76,77]. Fibrosis is often the final outcome of chronic inflammatory diseases, and develops from the simultaneous activation of multiple profibrotic pathways [75]. In normal tissue repair and healing response, fibroblasts within the injured tissue are transformed into myofibroblasts. In turn, myofibroblasts deposit ECM to aid in closing the wound and undergo apoptosis after the wound has healed [76]. However, in chronic injury and/or abnormal tissue repair, myofibroblasts persist and resist apoptosis.

OPN is critically involved in the fibroblast to myofibroblast transformation. Research has shown that OPN expression is required for TGFβ mediated myofibroblast differentiation and subsequent cardiac fibrosis [78]. In lung fibrosis and asthma, OPN is necessary for airway remodeling in response to chronic bronchial injury. This is, in part, due to OPN activating fibroblasts into myofibroblasts that orchestrate the repair process [79]. OPN expression is capable of inducing pulmonary fibrosis by initiating the migration, adhesion, and proliferation of fibroblasts through cytokine signaling and macrophage activation [80]. An abundant source of OPN in pulmonary fibrosis comes from epithelial cells which have undergone EMT—an alternative source of myofibroblasts in the microenvironment. In idiopathic pulmonary fibrosis, OPN induces fibrosis by increasing the expression of tissue inhibitor of metalloproteinase (TIMP) and type 1 collagen in fibroblasts and increasing the expression of the OPN activating protein matrix metallopretease 7 (MMP7) in alveolar epithelial cells [81].

Another area where OPN’s role in fibrosis is well-studied is the liver. The most common causes of liver fibrosis are chronic heptatitis B and C virus infection, alcohol abuse, primary biliary cirrhosis, primary sclerosing cholangitis, and NASH [82,83,84]. Hepatic stellate cells (HSCs) are the liver analogue to myofibroblasts in other regions of injury. In chronic liver disease (CLD), a greater response and proliferation is required and leads to an abnormal fibrogenic response—a process that is driven by HSCs. In models of chronic inflammation and hepatic fibrosis, OPN is upregulated in activated HSCs and further induces HSC activation [49,85]. Patients with alcoholic liver fibrosis have high levels of OPN in liver adipose tissue, and serum elucidating the clinical relevance of OPN in fibrosis. Interestingly, it has also been shown that OPN expression upregulates the expression of type 1 collagen and type 2 transforming growth factor-β receptor mRNA, suggesting that it not only promotes fibrosis but makes HSCs more sensitive to signaling molecules that promote fibrosis and EMT.

### 3.2. Activated Myofibroblasts Regulate EMT

Overall, OPN is able to activate fibroblasts to take on a myofibroblast (or activated HSC) phenotype. Myofibroblasts generated directly by OPN, or through other mechanisms, produce several key cytokines and secrete a myriad of signaling molecules including angiotensin II (Ang II), TNF-α, IL-6, sphingosine-1-phosphate (S1P), and TGFβ (Figure 2D) [86]. Each of these are proinflammatory factors with the ability to induce EMT programs in several different cell lines which result in fibrotic phenotypes. 

Ang II is able to activate Rho subfamily GTPases including RhoA, Rac1, and Cdc42 leading to downstream activation of key pathways that mediate fibrosis in cardiac myocytes [87]. One specific downstream consequence of Rho GTPase activation is cytoskeletal remodeling in epithelial cells—a hallmark of EMT [88]. Furthermore, inhibition of Ang II signaling, using the Angiotensin II type 1 blocker candesartan, can significantly reduce fibrosis by downregulating EMT [89].

Another cytokine increasingly recognized as a regulator of EMT is TNF-α. TNF-α, while usually associated with macrophages, is another secreted factor that can come from myofibroblasts and contributes to the fibrotic phenotype. In an *in vitro* model of kidney repair in response to injury, TNF-α induces renal epithelial cells to undergo EMT. In HK-2 cells exposed to TNF-α, Complement 3 (C3) is activated and E-cadherin expression repressed. Without TNF-α stimulation, HK-2 cells are unable to undergo C3-mediated EMT [90]. The role of TNF-α in renal fibrosis EMT may be related to the intermediate signaling molecule layilin, a c-type lectin-homologous protein that binds to cell surface cytoskeletal elements and is expressed in many organs. In a mouse model, TNF-α increases the expression of layilin. However, when layilin expression is inhibited, EMT does not occur even after TNF-α stimulation [91]. While this data supports TNF-α as an important component of EMT, there are additional elements that drive this response.

TGF-β is a major secreted factor from activated myofibroblasts and is a well-known effector of EMT, both in physiologic and pathologic settings. In fibrosis, TGF-β commonly exerts its effect on EMT through Smad, Notch, and EGFR intermediates [92]. Many TGF-β target genes are transcriptionally regulated by Smad3 or closely related complexes [93]. As a result, Smad family proteins can modify, repress, and induce EMT. Studies using Smad3 knockout mice demonstrate loss of EMT and subsequent fibrosis in lens, kidney, and liver injury models [94,95]. Notch signaling pathways are important in development, cell growth, and apoptosis with increasing evidence for a role in promoting fibrosis. In kidney fibrosis, inhibition of Notch signaling results in decreased fibrosis and prevents TGF-β mediated EMT [96]. Similarly, in a rat model, blockade of Notch signaling using a γ-secretase ameliorated TGF-β dependent EMT in both hepatocytes and HSCs [97]. Recently, studies have identified EGFR activation as an important TGF-β-related inducer of EMT. In an IPF cellular model, bronchial cells treated with TGF-β were shown to undergo EMT, which resulted in a pro-fibrotic phenotype. This process is highly dependent on activation of EGFR by TGF-β, identifying another way EMT occurs in epithelial cells [98].

### 3.3. Cancer-Associated Fibroblast Activation and Tumorigenicity

OPN is able to generate activated myofibroblasts that contribute to fibrosis across many organ systems. In cancer, the tumor microenvironment is populated by a similar cellular component, termed cancer-associated fibroblasts (CAF). CAFs (also called tumor-associated fibroblasts or carcinoma-associated fibroblasts) are derived from several sources including local fibroblasts, transformed tumors cells, and mesenchymal stem cells (MSCs) commonly recruited from the bone marrow [99]. OPN modulates tumor-specific EMT by generating CAFs (from both resident fibroblasts and recruited MSCs) which secrete a multitude of factors into the tumor microenvironment support tumor invasiveness and metastases [100].

Tumor-derived and exogenous OPN are both able to induce the transformation of MSCs to CAFs in breast cancer and liver cancer models. This occurs by stimulating MSCs to produce TGF-β creating a feedback loop that drives the CAF phenotype [101]. The transcription factor myeloid-zinc finger 1 (MZF1) is a critical intermediate to this process and activated through protein kinase A signaling [102]. In addition to MSCs, tumor-derived OPN is able to convert normal fibroblasts into CAFs. In both *in vitro* and *in vivo* breast cancer models, OPN secreted by tumor cells can promote the transformation of tissue-resident normal mammary fibroblasts into tumor-supporting CAFs [103].

### 3.4. Activated CAFs Regulate EMT

**Figure 3:**
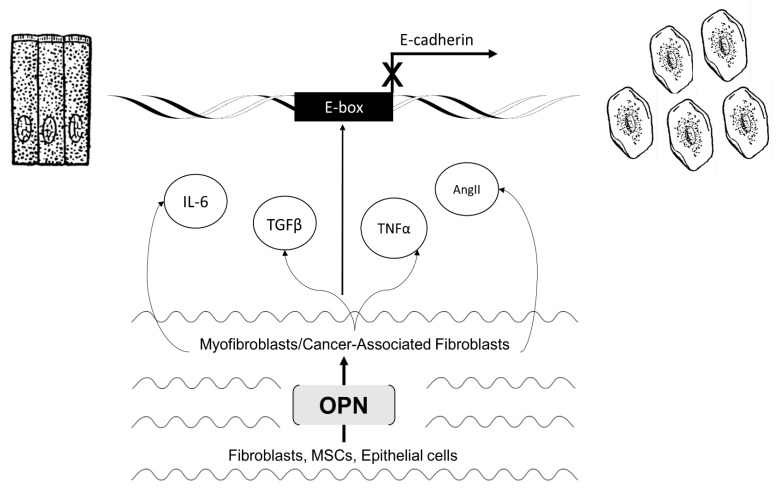
Microenvironmental regulation of EMT by OPN-mediated activation of stromal elements.

How OPN regulates EMT in tumors is largely a function of its ability to activate CAFs. CAFs, through paracrine interactions, are able to feedback to tumor cells using a diverse array of co-factors. This cross-talk serves many functions including the regulation of epithelial cell function, regulation of cellular response to inflammation, maintenance of environment homeostasis, promoting survival, and recruiting additional members of the stroma [99]. The following section will highlight the induction of EMT in various tumor cells in response to CAF-derived TGF-β and IL-6 (Figure 3).

TGF-β is secreted from the tumor stroma in many malignancies, largely sourced from CAFs [104]. CAF-derived TGF-β can induce EMT in several tumors via a multitude of mechanisms. Breast cancer cell lines undergo EMT in response to treatment with conditioned medium from CAFs isolated from invasive breast specimens. In this model, neutralizing antibody and small-molecule inhibition of TGF-β signaling prevents EMT in these cell lines [105]. TGF-β secreted by CAFs also can lead to EMT in bladder cancer cells, a process regulated by the long-coding RNA ZEB2NAT. Following the depletion of lncRNA-ZEB2NAT, conditioned media from CAFs no longer can cause EMT in human bladder cancer cell lines (5637, T24, and J82) [106].

TGF-β can also result in the formation of cancer stem cell populations within tumors—a process attributable to EMT. For example, TGF-β treatment of patient-derived colorectal tumor specimens results in upregulation of CD44+ stem cell populations and expression of N-cadherin. This suggests that TGF-β induces the formation of cancer stem cells through EMT [107]. In non-small cell lung cancer, TGF-β has a similar effect on cancer stemness [108]. Another intermediate for TGF-β-related acquisition of cancer stemness is miR-155 which, when overexpressed, results in EMT in liver cancer [109].

IL-6 is another key signaling molecule involved in EMT that can come directly from activated CAFs. In gastric cancer, IL-6 released by CAFs is able to modify the phenotype of tumor cells using IL-6/JAK/STAT3 signaling. Activation of this pathway results in tumor cell EMT and invasiveness, both *in vitro* and *in vivo* [110]. Further evidence of the role of IL-6 in regulating EMT comes from anti-IL-6 receptor assays in colon cancer. Antibody-mediated IL-6 receptor inhibition prevents angiogenesis by blocking the endothelial to mesenchymal transition (EndoMT) [111]. EndoMT is an extremely similar molecular process to that of EMT, however instead of epithelial cells acquiring a mesenchymal phenotype, endothelial cells undergo a transformation [112].

A more specific example of loss of EMT potential in the absence of IL-6 comes from pancreatic cancer. CAFs from pancreatic tumors treated with retinoic acid have decreased expression of IL-6. With downregulated IL-6, pancreatic tumor cells do not undergo EMT and do not gain an invasive phenotype [113]. Importantly, IL-6-related EMT may provide a mechanism to prevent chemotherapy resistance. In breast cancer, IL-6 from CAFs interacts with breast tumor cells to induce EMT and create a cancer stem cell subpopulation. This leads to resistance to tamoxifen, highly suggestive of a role for CAF-derived IL-6 in recurrence following conventional therapy [114].

## 4. Conclusions

The importance of OPN as a key regulatory factor of EMT is increasingly being recognized in both fibrosis and cancer. Understanding how OPN both directly and indirectly influences EMT in multiple settings and disease states can offer new targets for clinical therapeutics. Importantly, OPN is able to guide EMT through specific cellular signaling pathways and by restructuring the microenvironment to modify EMT programs. The shared molecular pathways between OPN and many traditional “master regulators” provides evidence that OPN is a major regulator of EMT.

## Figures and Tables

**Table 1:** Summary of current evidence linking osteopontin to epithelial-mesenchymal transition regulation.

Pathway	Model	Mechanism
Twist	Osteoblast-like	Twist upregulation causes OPN mRNA upregulation
	Breast Cancer	OPN/Twist/Bmi-1 pathway activates EMT programs
	Hepatocellular Carcinoma	OPN activates PI3K/AKT/Twist pathway leading to EMT
	Ovarian Cancer	OPN upregulates HIF-1α through PI3K/AKT pathway, upregulating Twist
	Gastric Cancer	OPN upregulates HIF-1 α through PI3K/AKT pathway, upregulating Twist
	Colorectal Cancer	High OPN secreting cell lines interact with Twist enhances metastasis
ZEB	Breast Cancer	OPN activates NFκB and increases ZEB1 and 2 to induce EMT
	Gastric Epithelial Cells	OPN activates NFκB, which transactivates ZEB1 to induce EMT in H. Pylori infection
	Liver Cells	OPN activates PI3K/pAkt/NFkB-signaling to cause liver fibrosis
	Hepatocellular Carcinoma	OPN interacts with p53, which upregulates miR-200 family to downregulate ZEB1 and ZEB2 to suppress metastasis
Snail	Breast Cancer	OPN expression cause EMT through overexpression of Snail
	Breast Cancer	OPN interacts with Runx2, Runx/Snail positive tumors exhibit EMT and increased malignancy
	Hepatocellular Carcinoma	OPN expression cause EMT through overexpression of Snail
	Skin Cancer	OPN and GLI1 are coregulated, GLI1 induces Snail1 and promotes EMT
	Brain Tumor	OPN and GLI1 are coregulated, GLI1 induces Snail1 and promotes EMT
	Nonalcoholic Steatohepatitis	OPN and GLI1 are coregulated, GLI1 induces Snail1 and promotes EMT
	Melanoma	OPN and GLI1 are coregulated, GLI1 induces Snail1 and promotes EMT
